# Systematic Approach to Find the Global Minimum of Relaxation Dispersion Data for Protein-Induced B–Z Transition of DNA

**DOI:** 10.3390/ijms22073517

**Published:** 2021-03-29

**Authors:** Kwang-Im Oh, Ae-Ree Lee, Seo-Ree Choi, Youyeon Go, Kyoung-Seok Ryu, Eun-Hee Kim, Joon-Hwa Lee

**Affiliations:** 1 Department of Chemistry and RINS, Gyeongsang National University, Gyeongnam 52828, Korea; orangekwang@gmail.com (K.-I.O.); dldofl24@naver.com (A.-R.L.); csr2915@nate.com (S.-R.C.); yuyun1948@naver.com (Y.G.); 2 Division of Magnetic Resonance, Korea Basic Science Institute, Ochang, Chungbuk 28119, Korea; ksryu@kbsi.re.kr; 3 Center for Research Equipment, Korea Basic Science Institute, Ochang, Chungbuk 28119, Korea; keh@kbsi.re.kr

**Keywords:** relaxation dispersion, CPMG, hZα_ADAR1_, Z-DNA, NMR, global search

## Abstract

Carr–Purcell–Meiboom–Gill (CPMG) relaxation dispersion spectroscopy is commonly used for quantifying conformational changes of protein in μs-to-ms timescale transitions. To elucidate the dynamics and mechanism of protein binding, parameters implementing CPMG relaxation dispersion results must be appropriately determined. Building an analytical model for multi-state transitions is particularly complex. In this study, we developed a new global search algorithm that incorporates a random search approach combined with a field-dependent global parameterization method. The robust inter-dependence of the parameters carrying out the global search for individual residues (GSIR) or the global search for total residues (GSTR) provides information on the global minimum of the conformational transition process of the Zα domain of human ADAR1 (hZα_ADAR1_)–DNA complex. The global search results indicated that a α-helical segment of hZα_ADAR1_ provided the main contribution to the three-state conformational changes of a hZα_ADAR1_—DNA complex with a slow B–Z exchange process. The two global exchange rate constants, *k_ex_* and *k_ZB_*, were found to be 844 and 9.8 s^−1^, respectively, in agreement with two regimes of residue-dependent chemical shift differences—the “dominant oscillatory regime” and “semi-oscillatory regime”. We anticipate that our global search approach will lead to the development of quantification methods for conformational changes not only in Z-DNA binding protein (ZBP) binding interactions but also in various protein binding processes.

## 1. Introduction

Molecular motions play important roles in biological processes; however, their complexity makes the accurate identification of motions difficult. NMR spectroscopy is a powerful tool that had been used to characterize these motions according to the chemical exchanges and kinetic processes of molecules [1,2,3]. In particular, Carr–Purcell–Meiboom–Gill (CPMG) relaxation dispersion (RD) experiments can be used to provide information on protein folding and binding events, with protein motions reported in an ms-to-μs timescale [4,5,6,7,8].

The first step of CPMG analysis is the establishment of an appropriate model to determine the exchange process. The two-state exchange process is a simple kinetic model that has been widely used to understand molecular motions, such as enzyme catalysis, protein folding, molecular recognition, and allostery, using CPMG RD analysis [1,9,10,11,12]. Despite the fact that multi-state conformational exchanges including three-state transitions have also been studied using CPMG RD [13,14,15,16,17], elucidating the dynamics of more complicated motions of multi-state exchange processes with high accuracy remains challenging.

Here, we provide a systematic procedure to obtain accurate dynamics parameters and chemical shifts from CPMG-based experimental data. Our approach is to generate an array of parameters using a random search method combined with various constraints. The R_2_ profiles are numerically computed by a three-state conformational transition model that includes fast and slow processes. In the proposed rationalization strategy and hierarchical steps, we provide a set of parameters for finding the exact global minimum according to the constraints. In principle, the parameterization is the direct mapping of multi-state transitional variables in the pseudo-three state transition model using spatial combinations to find the minimum χ^2^ value.

**Figure 1:**
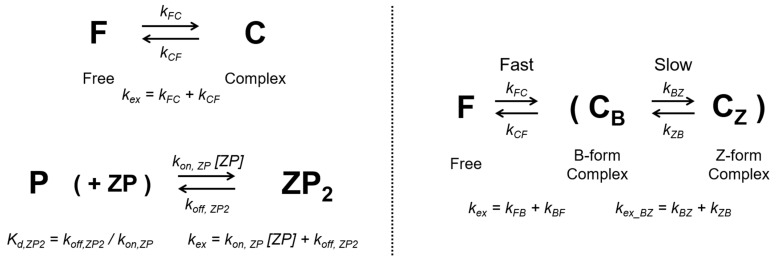
Two types of conformational transition model and ^15^N Carr–Purcell–Meiboom–Gill (CPMG) relaxation dispersion of hZα_ADAR1_ bound to d(CG)_3_: Two-state models of association and dissociation of protein ligand complex (upper) and hZα_ADAR1_−DNA complex (lower) (**Left**); Pseudo-three-state model of association and dissociation process in the protein–ligand complex composed of an independent two-state transition between B-form and Z-form in the complex (**Right**).

We applied this novel approach to identify the protein-induced B–Z conformational transition of DNA in a Z-DNA binding protein (ZBP) and DNA complex [18,19,20,21,22]. A left-handed helical Z-DNA structure can form from ZBPs, including human double-stranded (ds) RNA-specific adenosine deaminase I (ADAR1) [23,24], the DNA-dependent activator of IFN-regulatory factor (DAI, also known as DLM-1, ZBP1) [25,26], poxviral E3L protein [27,28], and RNA-dependent protein kinase (PKZ) [29]. In general, the intermolecular interactions of ZBPs and Z-DNA are mediated by residues in the third helix and the β-hairpin region [13,22,30]. Structural studies of the Zα domain of human ADAR1 (hZα_ADAR1_) and a nucleic acid complex have shown protein-induced B–Z or A–Z transitions of DNA or RNA, respectively [22,31]. More recently, it has been reported by imino proton and heteronuclear single quantum correlation (HSQC) titrations that the rate of the A–Z RNA transition is 30-times slower than the B–Z structural transition of DNA induced by hZα_ADAR1_ [13]. Therefore, the proposed multi-state conformational changes of hZα_ADAR1_ and DNA complex indicate that the model is suitable to determine R_2_ profiles based on multi-magnetic field CPMG profiles. The suggested conformational transitions are described in Figure 1.

## 2. Results

The dynamic motion of hZα_ADAR1_ induced by conformational transitions on a ms-to-μs timescale was investigated by modeling using the site-specific CPMG RD profiles. We used a hierarchical approach to characterize the quantitative kinetic and chemical shift parameters undergoing the three-state conformational transition: (i) Residues representing the three-state conformational exchanges were determined by error of fitting using the two-state model; (ii) the residue dependent R_2_ profiles were numerically computed by random parameters constrained by chemical shift values considering two different magnetic fields; and (iii) re-constrained kinetic and chemical shifts parameters were applied to characterize the global conformational transition through the entire backbone of hZα_ADAR1_.

### 2.1. Classification of Three-State Conformational Transition of hZα_ADAR1_

**Figure 2:**
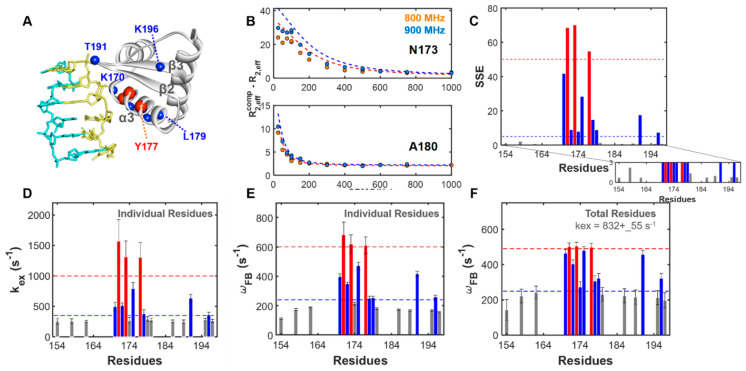
Fitting results of Carr–Purcell–Meiboom–Gill (CPMG) relaxation dispersion (RD) profiles using two-state model: (**A**) Mapping the CPMG RD results on the hZα_ADAR1_ structure (PDB ID: 1QBJ); (**B**) ^15^N CPMG RD data for two representative residues: (top) Asn173 (a case for three-state transition) and (bottom) Ala180 (a case for two-state transition); (**C**) sum of squared errors (SSE) plots in fitting to the two-state model; (**D**) the exchange rate constants from individual fitting with the two-state model, as described in Equation (2); (**E**) chemical shifts between free and complex states from individual fitting with the two-state model; (**F**) chemical shifts from global fitting with the two-state model, which includes rate constant, *k_ex_*, as a shared parameter. The individual and global fitting parameters are presented in Table S1.

The rate constants for association and dissociation of hZα_ADAR1_ onto d(CG)_3_, *k_FB_* and *k_BF_*, were determined by profiles of ^15^N amide CPMG RD data. In the previous study, the two major conformational transitions between free ***P*** and complex ***ZP_2_*** were obtained in the range of [N]_t_/[P]_t_ ≪ 0.5. The [N]_t_ and [P]_t_ are total concentrations of the DNA duplex and hZα_ADAR1_, respectively [32]. Thus, we initially analyzed CPMG profiles based on a two-state model in the fast limit, as shown in Figure 1 and Figure 2 (also see Figure S1 and Table S1). The schemes of the two-state and pseudo-three state models of the conformational transition are described in Figure 1, respectively. In the two-state model, the ***P*** and ***ZP_2_*** states are considered free and a complex of hZα_ADAR1_, respectively. Association and dissociation rate constants are given as *k_FB_* = *k_on,ZP_* × [*ZP*] and *k_BF_* = *k_off,ZP2_*, respectively. The differences in the transverse relaxation rate constant between the complex and the free state were fitted to Equation (2). The global fit result of the rate constant, *k_ex_*, is 832 s^−1^ (Table 1). The sum of squared errors (SSE) and fitting parameters were used to classify the residues of hZα_ADAR1_ into two groups following the two-state or three-state conformational transition model. Cases with oscillatory CPMG profiles due to slow motion show relatively larger chemical shift differences in the free to complex conformational transition. In the following section, we present a method to determine the site-specific dynamics of 11 of the 19 residues showing oscillatory profiles.

### 2.2. Site-Specific Analysis of Three-State Model Using Field-Dependent CPMG Profiles

**Figure 3:**
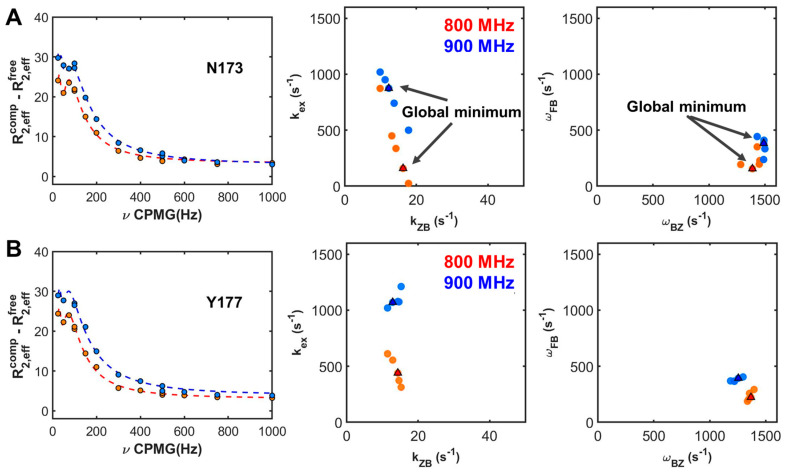
^15^N CPMG relaxation dispersion data (left), residue dependent global rate constants (middle), and residue dependent chemical shifts (right) for Asn173 (**A**) and Tyr177 (**B**) as representative residues. Dashed lines indicate $R_{2}^{Calc}$ using global search for individual residues (GSIR) for single-field results, 800 MHz (red or orange) and 900 MHz (blue or pale blue). Global parameters are shown with squares, while the optimized parameters of re-sampled results are shown with closed circles. All of the results are presented in Table S2.

Single-field numerical calculations were performed for the three-state conformational transition consisting of an independent two-state transition, as described in Figure 2B. Minimization of re-sampled parameters was carried out from one set of the χ^2^ surface. Comparing χ^2^ values from $R_{2}^{Calc}$ of re-sampled results provided the global minimum χ^2^ value, *R_02_*, *k_ex_*, *k_ZB_*, Δ*ω_FC_*, and Δ*ω_BZ_*. In Figure 3, residue-dependent parameters in different magnetic fields are described (see also Figures S2–S4 and Table S2). In principle, parameters with the minimum χ^2^ values between 800 MHz and 900 MHz data should be identical. In the current global search for individual residues using a single magnetic field-based approach, relative trends between residue-dependent parameters show consistency at different magnetic fields; however, the complexity of the multi-state conformational transition leads to discrepancies in the absolute values of parameters among the different magnetic field data (Figure S5). These discrepancies indicate that single-field data are insufficient to accurately characterize quantifiable kinetic parameters.

To increase the validity of the GSIR method, it adopts multiple static magnetic fields with restrained variables of chemical shift, Δ*ω_FC_* and Δ*ω_BZ_*. The proportional correlation between chemical shifts in different magnetic fields validates and reduces the number of parameter arrays during numerical calculations. Finally, in parallel with field-dependent parameterization, the Monte Carlo approach provides more plausible absolute values for GSIR.

**Figure 4:**
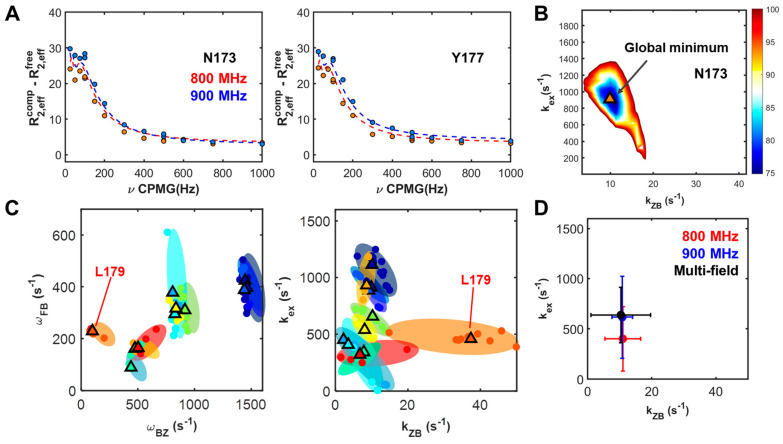
GSIR results are described: (**A**) ^15^N CPMG RD data for two representative residues, Asn173 and Tyr177 with $R_{2}^{Calc}$ lines based on GSIR; (**B**) representative plot of the global χ^2^ map for rate constants; (**C**) optimized chemical shifts (left) and rate constants (right) with the minimized χ^2^ of re-sampled results (closed circles) and global minima (triangles); (**D**) averaged residue-dependent rate constants for single-field and multi-field GSIR results. Different residues are indicated by different colors, while shaded ellipses indicate the areas of minimized χ^2^s of re-sampled results.

Figure 4 presents the global χ^2^ map and GSIR results (Figures S2, S6, and S7, and Table 2). The minimized values for re-sampled sets are represented together with the optimized values of parameters at the global point in the χ^2^ map. The $R_{2}^{Calc}$ profiles based on the minimum χ^2^ show good agreement with the experimental CPMG profiles, as plotted in Figure 4 and Figure S2. The dynamics parameters for calculating the global minimum of χ^2^ are shown in Figure 3C,D. The residues, Glu171, Asn173, and Tyr177 reveal the greatest change in chemical shift due to the slow exchange motions of the B–Z conformational transition in the complex. Moreover, the exchange rate constant, *k_ex_*, at a value of 638 s^−1^, is broadly distributed through individual residues. The *k_ZB_*, at 10 s^−1^, also exhibits a broad distribution due to the high value of *k_ZB_* at the residue, Lys 179. Removing the large value of *k_ZB_* at the Lys 179 residue can result in a narrower distribution.

### 2.3. Dependencies of Parameters: Global Search for Total Residues (GSTR)

**Figure 5:**
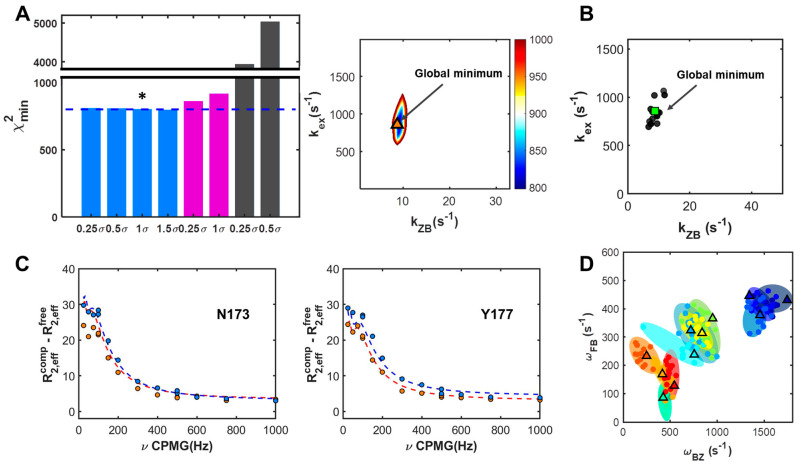
GSTR results are described: (**A**) Comparisons of χ^2^ minima under the different restraints (left), and the representative global χ^2^ map with minimum value of rate constant as indicated by asterix, * (right); (**B**) residue dependent optimized rate constants with minimized χ^2^ of re-sampled results (closed circles) and global minima (triangles); (**C**) ^15^N CPMG relaxation dispersion data for two representative residues, Asn173 and Tyr177 with $R_{2}^{Calc}$ lines based on GSTR; and (**D**) residue dependent optimized chemical shifts with minimized χ^2^ of re-sampled results (closed circles) and global minima (triangles). Different residues are indicated by different colors, while shaded ellipses indicate the areas of re-sampled results. * *p* < 0.05.

GSTR was performed to obtain rate constants and chemical shifts associated with the three-state conformational transition induced by protein binding onto DNA. To sample the valid $R_{2}^{Calc}$ values, various randomized parameters were constrained using Gaussian distributions. In brief, the unconstrained GSTR approach does not achieve convergence of χ^2^, so to determine the limitation condition of parameterization, we tested various Gaussian distribution widths of rate constants and chemical shifts based on the standard deviation of rate constants, the mean value of rate constants, and the middle values of chemical shift (Figures S8 and S9). These constraint ranges were obtained from the GSIR analysis results. Figure 5A shows comparisons of the minimum χ^2^, which represents similar minimized χ^2^ values for the cases where the range of Gaussian distribution widths of the chemical shifts for the global search process is 25%. The similarity of the minimized χ^2^ values indicates that in the GSIR results, a distribution range lower than 1.5 σ of the mean value is sufficient to accurately calculate the parameters of the three-state conformational transition. $R_{2}^{Calc}$ profiles were generated and show great agreement with the experimental observations of CPMG profiles (see also Figure S10). The globally optimized rate constants, *k_ex_* and *k_ZB_*, were 844 and 9.8 s^−1^, respectively, as shown in Table 2.

## 3. Discussion

Our results provide a systematic approach to map the multi-state conformational transition on a computational basis with rational constraints of input and numerical calculation limits. In order to determine quantitative kinetic and chemical shift parameters, a three-step strategy was applied. First, residues containing the contributions of slow motions in the three-state conformational transition were grouped based on the mismatches between CPMG profiles and fitting lines using a two-state transition model in the fast limit. Second, we used a Monte Carlo approach, with simultaneous constraint of the multi-magnetic field CPMG profiles and chemical shift parameters to identify totals of the residue-based variables, *R_02_*, *k_ex_*, *k_ZB_*, Δ*ω_FC_*, and Δ*ω_BZ_*. Finally, numerical parameter mapping to determine the global rate constants and residue-dependent chemical shifts was performed based on the global search results of individual residues obtained in the second step.

**Figure 6:**
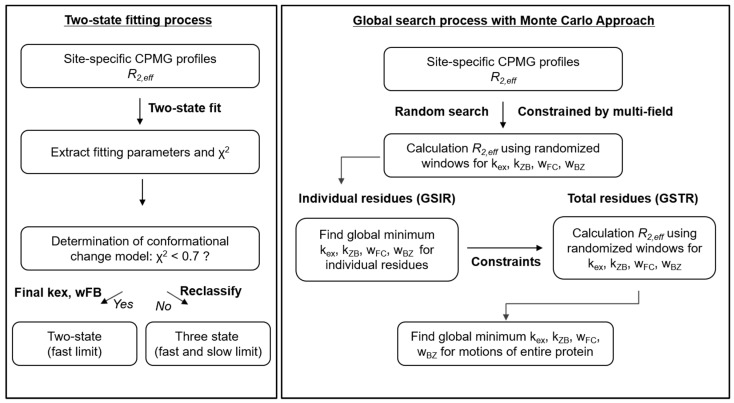
Flow chart of the two-state fitting and the global search process for analyzing CPMG profiles of hZα_ADAR1_ and the hZα_ADAR1_–DNA complex.

To determine global rate constants for the three-state conformational changes, various constraints were applied, since parameterization without constraints did not provide globally minimized $R_{2}^{Calc}$ profiles. Before computing the GSTR results, the GSIR parameters provided sufficient information to generate the input variables. Considering Gaussian distributions of rate constants and residue-dependent chemical shifts in relation to shared chemical shifts for multi-state profiles allows for well-optimized parameterization with high accuracy without the need for long-duration computational simulations. Residue-dependent global search constrained by independently measured CPMG RD profiles of multi-magnetic field results shows different value and narrower distribution of the exchange kinetic constant during the conformational transition between free to bound states than the cases from CPMG RD profiles of the single-magnetic field results. Also, GSTR process re-constrained by residue-dependent GSIR results provides the information on the entire changes of protein motions during three-state conformational changes. Taken together, the computational algorithm with the global search process is presented in Figure 6.

As shown in Figure 5 and Table 2, the cases with high oscillatory contributions to CPMG profiles were found to have large values of Δ*ω_BZ_*. The cases with residues Glu171, Asn173, and Tyr177 are those that exhibit the sum of squared errors (SSE) in fitting to the two-state model. Based on the global analysis of the CPMG profiles to characterize the protein-induced conformational exchanges of the B–Z transition in DNA, we suggest three-state conformational changes including two independent two-state exchange processes: (i) The fast exchange between the free and DNA-complex state of hZα_ADAR1_; (ii) the slow exchange associated with the B–Z transition of DNA in the hZα_ADAR1_–DNA complex (Figure 2B). Interestingly, the cases with high oscillatory contributions to CPMG profiles were found to have large values of Δ*ω_BZ_* on the three residues, Glu171, Asn173, and Tyr177, were found to provide the main contributions to the slow exchange process in CPMG profiles (Figure 5 and Table 2). These residue characteristics indicate the presence of two distinct regimes—the “dominant oscillatory regime” and “semi-oscillatory regime”.

We anticipate that this machine-learning-type approach will provide new insight to understand biomolecular motions in conformational transitions of protein–protein or protein–nucleic acid complexes.

## 4. Materials and Methods

Here we provide the experimental methods and the global search algorithms. One of the most common methods for RD curve fits is the grid search approach, but it does not always provide the global minimum, which imparts the best-fit and parameters. Moreover, increasing grid size is not the best way to improve the quality of fits as it increases the computational time. This is related to a common issue of the local minimum problem, which occurs in a non-linear curve fitting process. The same local minimum issue arises during the CPMG profile fitting process. For this reason, in practice, the development of a new logical process to find the global minimum is necessary to characterize the multi-state conformational transition by modeling CPMG profiles. Particularly, hierarchical step of Monte Carlo approach with randomized parameterization with rational constraints was employed for the robust determination of the global χ^2^ values.

### 4.1. Sample Preparation

The DNA oligomer was purchased from M-biotech Inc (Seoul, Korea). This DNA oligomer was purified by reverse-phase HPLC and desalted with a Sephadex G-25 gel-filtration column. The amount of DNA was represented as the concentration of the double-stranded sample. The hZα_ADAR1_ (E140−Q202) was cloned into E. coli expression plasmid pET28a (Novagen, WI, USA). To produce ^15^N labeled hZα_ADAR1_, BL21(DE3) bacteria were grown in M9 medium that contained 1 g/L ^15^NH_4_Cl. The details of purification and expression of ^15^N-labeled hZα_ADAR1_ proteins were described in a previous study [1]. The DNA and protein samples were dissolved in a 90% H_2_O/10% D_2_O NMR buffer containing 10 mM sodium phosphate (pH 6.0) with 100 mM NaCl salt.

### 4.2. CPMG RD Results

The ^15^N amide CPMG RD experiments were performed using free ^15^N-labeled hZα_ADAR1_ and ^15^N-labeled hZα_ADAR1_ complexed with d(CG)_3_ at 35 °C. RD profiles of ^15^N amide of hZα_ADAR1_ were measured in two magnetic fields on a Bruker Avance-III 800-MHz NMR spectrometer or a Bruker Avance-III 900-MHz spectrometer (KBSI, Ochang) equipped with a cold probe. The constant time interval, *T_relax_*, was 60 ms and 14 values of *υ_CPMG_* (=1/(2*τ_CP_*)) in the range 25–1000 Hz were used in all the experiments. Transverse relaxation rates *R_2,eff_* from each cross-peak signal at each value were calculated using the following equation:

(1) $$R_{2,eff}\left(\nu_{CPMG}\right)=\frac{1}{T}ln\left\{\frac{I_{I}\left(\nu_{CPMG}\right)}{I_{0}}\right\}$$

where *I(υ_CPMG_*) and *I*_0_ are the peak intensity at a given value of *υ_CPMG_* with delay time of 60 and 0 ms, respectively.

### 4.3. Binding Models and Global Searches

Classification: ^15^N amide CPMG RD profiles were initially fit to the two-state model, as described in Figure 2. The non-linear least-squares fitting algorithm was used and implemented in the MATLAB 2020a package (MathWorks Inc., Natick, MA, USA). For the fast exchange limit, all kinetic parameters calculated using the following equation:

(2) $$\begin{array}{ll} R_{2,eff}^{comp}\left(\nu_{CPMG}\right)- & R_{2,eff\;}^{free}\left(\nu_{CPMG}\right)\\ & =\;R_{2}^{0}+\;\frac{P_{F}P_{C}{\left(\Delta \omega_{FC}\right)}^{2}}{k_{ex}}\left\{1-\frac{4\nu_{CPMG}}{k_{ex}}\tanh\left(\frac{k_{ex}}{4\nu_{CPMG}}\right)\right\} \end{array}$$

where *P_F_*, *P_C_*, Δ*ω_FC_*, and *k_ex_* are the relative populations of the free and complex states, chemical shift changes between two states, and exchange rate constant, respectively. The sum of squared errors (SSE) was selected as the classification parameter for the conformational transition model, as described in Figure 2. The global fitting method was applied to examine the global exchange rate constant and residue-dependent chemical shifts between the free and complex states (see Figure 2F). In addition, residues with SSE greater than 0.7 were analyzed according to the three-state kinetic model, as described in following equation:

(3) $$\begin{array}{ll} R_{2,eff}^{comp}\left(\nu_{CPMG}\right)- & R_{2,eff\;}^{free}\left(\nu_{CPMG}\right)\\ & =R_{2}^{0}+\;\frac{P_{F}P_{C}{\left(\Delta \omega_{FC}\right)}^{2}}{k_{ex}}\left\{1-\frac{4\nu_{CPMG}}{k_{ex}}\tanh\left(\frac{k_{ex}}{4\nu_{CPMG}}\right).\right\}\\ & +k_{ZB}\left\{1-\frac{\sin\left(\Delta \omega_{BZ}/4\nu_{CPMG}\right)}{\Delta \omega_{BZ}/4\nu_{CPMG}}\right\} \end{array}$$

The *k_ZB_* and Δ*ω_FC_* are the exchange rate constant and chemical shift difference between the *C_B_*–*C_Z_* states, respectively.

Global Search Algorithm: Global search algorithms were performed to find the global minimum of the three-state conformational transition using the global exchange rates, *k_ex_* and *k_ZB_*, and residue-dependent chemical shifts, Δ*ω_FC_* and Δ*ω_BZ_*. At the independent fast and slow limits, CPMG RD profiles were numerically calculated using Equation (3). $R_{2}^{Calc}$ was computed using the sum of all $R_{2}^{Calc}$s on a per-residue basis to obtain the χ^2^ function, as described in Equation (4). below,

(4) $$\chi_{total}{}^{2}\left(R_{2}^{0},k_{ex},k_{ZB},\Delta \omega_{FC},\Delta \omega_{BZ}\right)=\sum {\left(R_{2}^{calc}\left(R_{2}^{0},k_{ex},k_{ZB},\Delta \omega_{FC},\Delta \omega_{BZ}\right)-R_{2}^{exp}\right)}^{2}$$

The global χ^2^ values were estimated using the Monte Carlo approach, where the randomized χ^2^ surfaces were numerically obtained on summation profiles based on the bootstrap procedure using sub-arrays of parameters, *R_02_*, *k_ex_*, *k_ZB_*, Δ*ω_FC_*, and Δ*ω_BZ_*. The Monte Carlo approach was performed in the MATLAB 2020a package. Initial kinetic parameters, *R_02_*, *k_ex_*, *k_ZB_*, Δ*ω_FC_*. and Δ*ω_BZ_*, were randomly generated within the range of values obtained from the two-state fit ±0.2, 1–2000 s^−1^, 1–50 s^−1^, 1–1500 Hz, and 1–1500 Hz, respectively, for the 11 selected residues. Five arrays of re-sampled parameters were extracted to compute the minimum χ^2^ from one set of randomized χ^2^ surface. GSIR determined global minimum parameters using a global search on a per-residue basis, while GSTR was performed to determine the global rate constants and residue-dependent chemical shifts. For GSTR, initial kinetic parameters, *k_ex_*, *k_ZB_*, Δ*ω_FC_*, and Δ*ω_BZ_*, were generated from the Gaussian distributed random numbers to determine the constraints to be implemented for the numerical calculations. A rational, step-by-step, hierarchical approach was used to find appropriate constraints of the dynamic parameters throughout the entire residue and this approach described in the next section.

Constraints: For GSIR, chemical shift parameters Δ*ω_FC_* and Δ*ω_BZ_*, a strong inter-dependency between two magnetic fields, $\Delta \omega_{FC}^{i}=\;j/i(\Delta \omega_{FC}^{j})$ and $\Delta \omega_{BZ}^{i}=\;j/i(\Delta \omega_{BZ}^{j})\;$was applied, where *i* and *j* are the magnetic fields. Kinetic rate constants between two magnetic fields, $k_{ex}^{i}=\;k_{ex}^{j}$ and $k_{ZB}^{i}=\;k_{ZB}^{j}$, were used, since kinetic rate constants have to be equal under the different magnetic fields.

During the GSTR process, global parameters did not converge during calculation of the global minimum based on randomized global rate constants and residue-dependent chemical shifts (Figures S8 and S9). As a first step, the initial values of the rate constants were chosen from a range of standard deviations, σ, calculated from the mean value of GSIR residue-dependent rate constants, as shown in Table 1 and Table 2, using Gaussian distributions. Optimization of the suppressed rate constant did not lead to convergence of the χ^2^ values, so the chemical shift difference was constrained to obtain plausible values of residue-dependent chemical shifts. As the second step, to obtain more flexible boundaries for chemical shift screening rate constants, a broad range of constraints for chemical shifts with Gaussian distributions was applied. A 25% deviation from the middle value of residue-dependent chemical shifts was used for the Gaussian distributed width along with various Gaussian distributed rate constants (see Figure S9). The minimum χ^2^ was determined using various standard deviations (0.25σ, 0.5σ, σ, and 1.5σ) of the rate constants simultaneously with the chemical shift constrained. Finally, GSTR provided rational results of the globally optimized kinetic parameters (Table 2).

## Figures and Tables

**Table 1:** Conformational exchange rate constants extracted by different methods are described.

	*k_ex_* (s^−1^)	*k_ZB_* (s^−1^)
Two-state	832 ± 55	-
GSIR	638 ± 277	10 ± 9
GSIR (800 MHz)	401 ± 318	11 ± 5.5
GSIR (900 MHz)	615 ± 408	11 ± 3.2
GSTR ^1^	844 ± 41 (858)	9.8 ± 0.8 (8.7)

^1^ Averaged values of rate constant with constraints *k* ± 0.25σ to 1.5σ and Δ*ω* ± 0.25x <Δ*ω*> (rate constant for the case with constraints: *k* ± σ and Δ*ω* ± 0.25x <Δ*ω*>).

**Table 2:** GSIR and global search for total residues (GSTR) results are presented. The chemical shifts were shown as the 800 MHz field cases. For GSTR: Averaged values of rate constant with constraints k ± 0.25σ to 1.5 σ and Δ*ω* ± 0.25x <Δ*ω*> (rate constant for the case with restraints: k ± σ and Δ*ω* ± 0.25x <Δ*ω*>).

	GSIR				GSTR			
Residue	*k_ex_* (s^−1^)	Δ*ω_FB_* (Hz)	*k_ZB_* (s^−1^)	Δ*ω_BZ_* (Hz)	*k_ex_* (s^−1^)	Δ*ω_FB_* (Hz)	*k_ZB_* (s^−1^)	Δ*ω_BZ_* (Hz)
K170	450	378	2.25	807	845 ± 41.3 (858)	342 ± 14.0 (320)	9.81 ± 0.75 (8.74)	633 ± 62.1 (718)
E171	1110	424	10.1	1450		385 ± 36.3 (430)		1590 ± 145 (1740)
I172	407	295	3.56	827		226 ± 22.9 (238)		758 ± 83.9 (755)
N173	912	396	9.93	1460		407 ± 31.6 (446)		1473 ± 128 (1340)
R174	343	89.3	7.86	440		85.8 ± 16.0 (85.9)		419 ± 45.4 (424)
V175	656	310	10.3	915		345 ± 14.1 (366)		900 ± 79.7 (951)
Y177	885	388	9.29	1440		367 ± 45.8 (378)		1404 ± 165 (1450)
S178	932	161	8.62	474		143 ± 19.0 (169)		456 ± 54.5 (416)
L179	458	229	37.4	99.5		264 ± 23.3 (233)		176 ± 53.6 (248)
T191	538	316	8.08	836		334 ± 27.7 (314)		764 ± 90.1 (838)
K196	321	164	6.79	503		144 ± 19.5 (128)		483 ± 89.6 (542)

## Data Availability

The data presented in this study are available on request from the corresponding author.

## Supplementary Materials

The following are available online at https://www.mdpi.com/article/10.3390/ijms22073517/s1, Figure S1: The ^15^N CPMG RD data and the global fits to two-state model, Figure S2: The ^15^N CPMG RD data and GFIR results, Figure S3: Residue dependent rate constants, Figure S4: Residue dependent chemical shifts, Figure S5: Residue dependent GSIR results for all residues, Figure S6: Global χ^2^ maps projected onto rate constants for GSIR results, Figure S7: Global χ^2^ maps projected onto chemical shifts for GSIR results, Figure S8: Global χ^2^ maps projected onto rate constants for GSTR, Figure S9: Histograms of constrained input parameters, Figure S10: The ^15^N CPMG RD data and GFTR results, Table S1: Conformational exchange rate constants and chemical shift using two-state model, Table S2: Conformational exchange rate constants and chemical shift using single-field basis GSIR.

## Abbreviations

ADAR1	RNA-specific adenosine deaminase I
CPMG	Carr–Purcell–Meiboom–Gill
HSQC	Heteronuclear single quantum correlation
hZα_ADAR1_	The Zα domain of human ADAR1
GSIR	Global search for individual residues
GSTR	Global search for total residues
RD	Relaxation dispersion
SSE	Sum of squared errors
ZBP	Z-DNA binding protein
