# Supplementary material for: Systematic Approach to Find the Global Minimum of Relaxation Dispersion Data for Protein-Induced B–Z Transition of DNA
Source: Int J Mol Sci. 2021 Mar 29;22(7):3517. doi: 10.3390/ijms22073517 (PMC8037647; doi:10.3390/ijms22073517)
Supplement: Supplementary file 1 [file ijms-22-03517-s001.pdf]

Supplementary Material

Systematic approach to find the global minimum of relaxation dispersion data for protein-induced B-Z transition of DNA

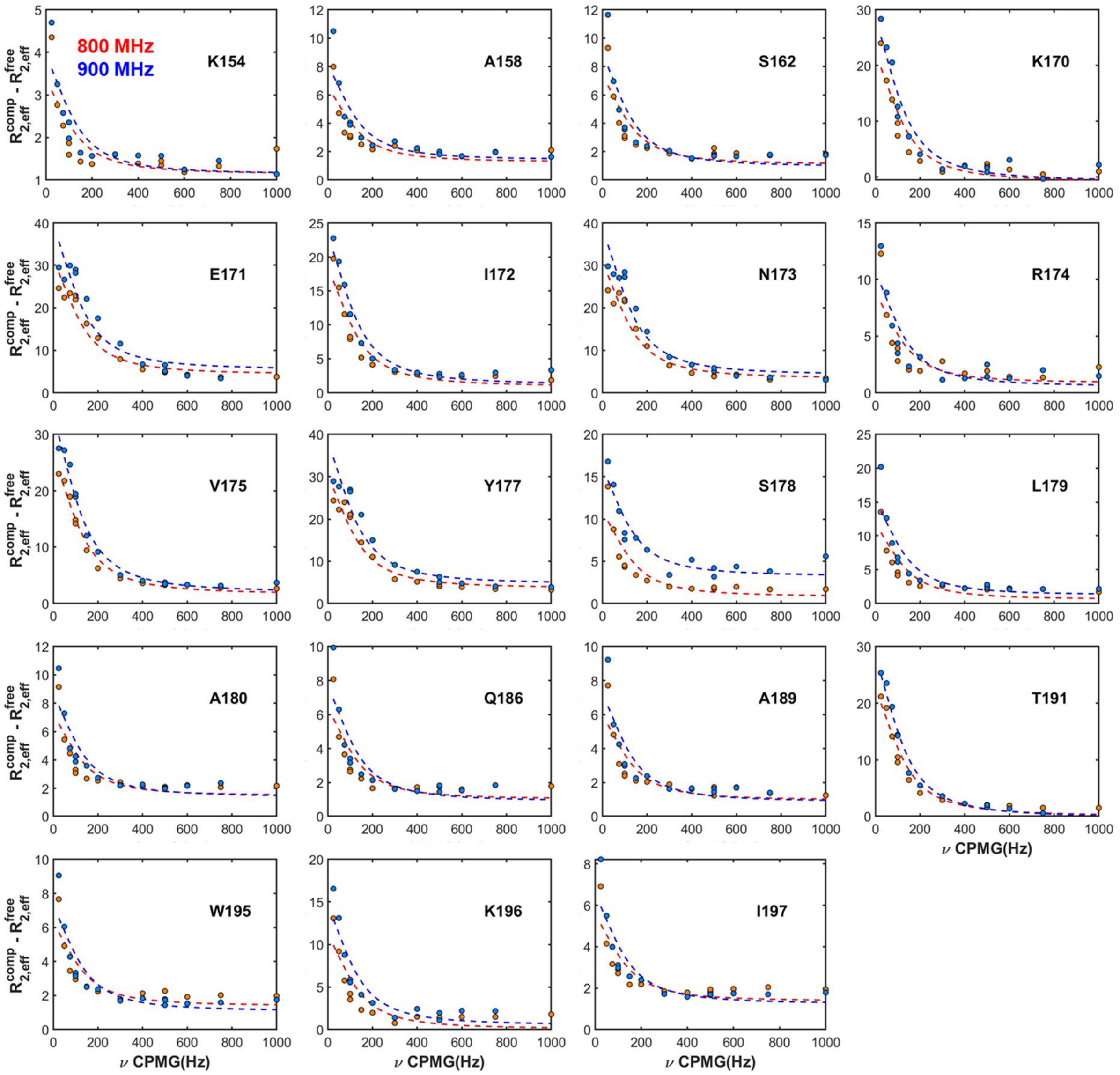

Figure S1. The ^{15}N CPMG relaxation dispersion data (circles) and the best global fits to two-state model are plotted. 800 MHz and 900 MHz results are indicated by red and blue colors, respectively. Data were fitted to Eq. 2 in the main text. Name of residues are indicated.

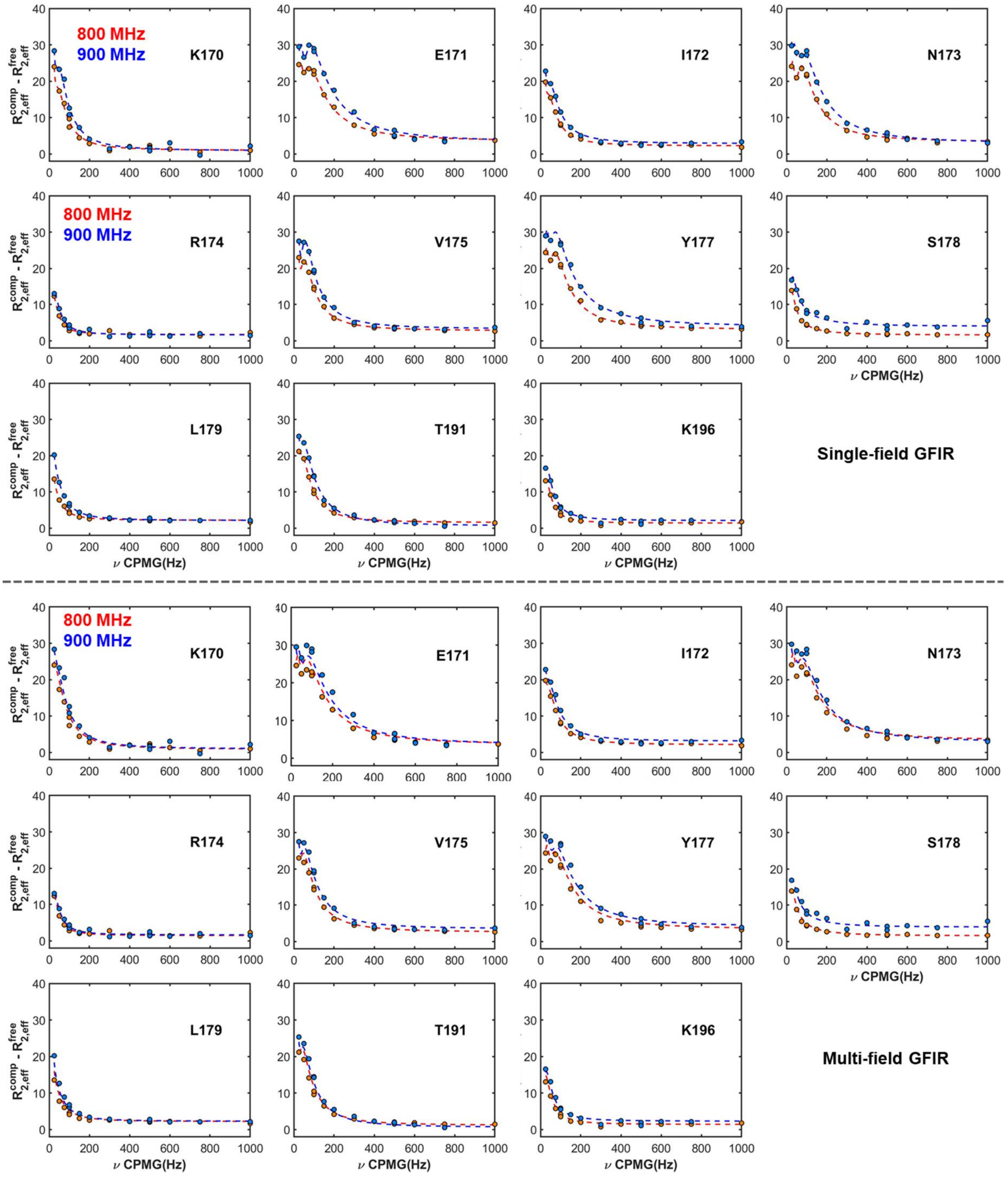

Figure S2. The ^{15}N CPMG relaxation dispersion data and R_2^{Calc} lines of global search for individual residues (GFIR) for single-field results (upper) and multi-field results (lower) of the d(CG)$_3$-hZα_{ADAR1} complexes. The best GSIR results to Eq. 3 with 800 MHz and 900 MHz data are described by red and blue lines, respectively. Names of residues are denoted.

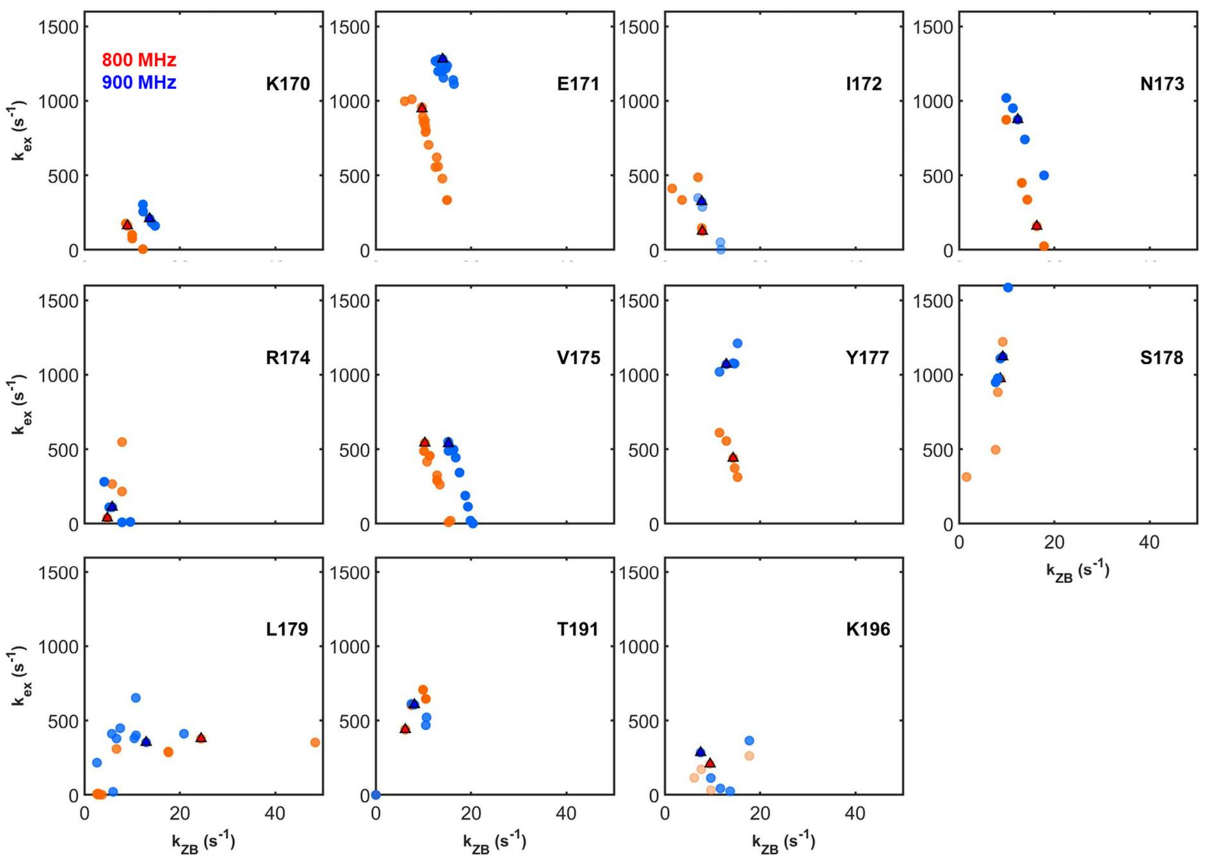

Figure S3. Residue-dependent rate constants, k_{ex} and k_{ZB}, and minimum χ^2 are plotted. Global parameters are described by squares, while the optimized parameters of re-sampled results are shown with closed circles. All values of results are presented in Table S2. Results for 800 MHz and 900 MHz are indicated by red and blue colors, respectively.

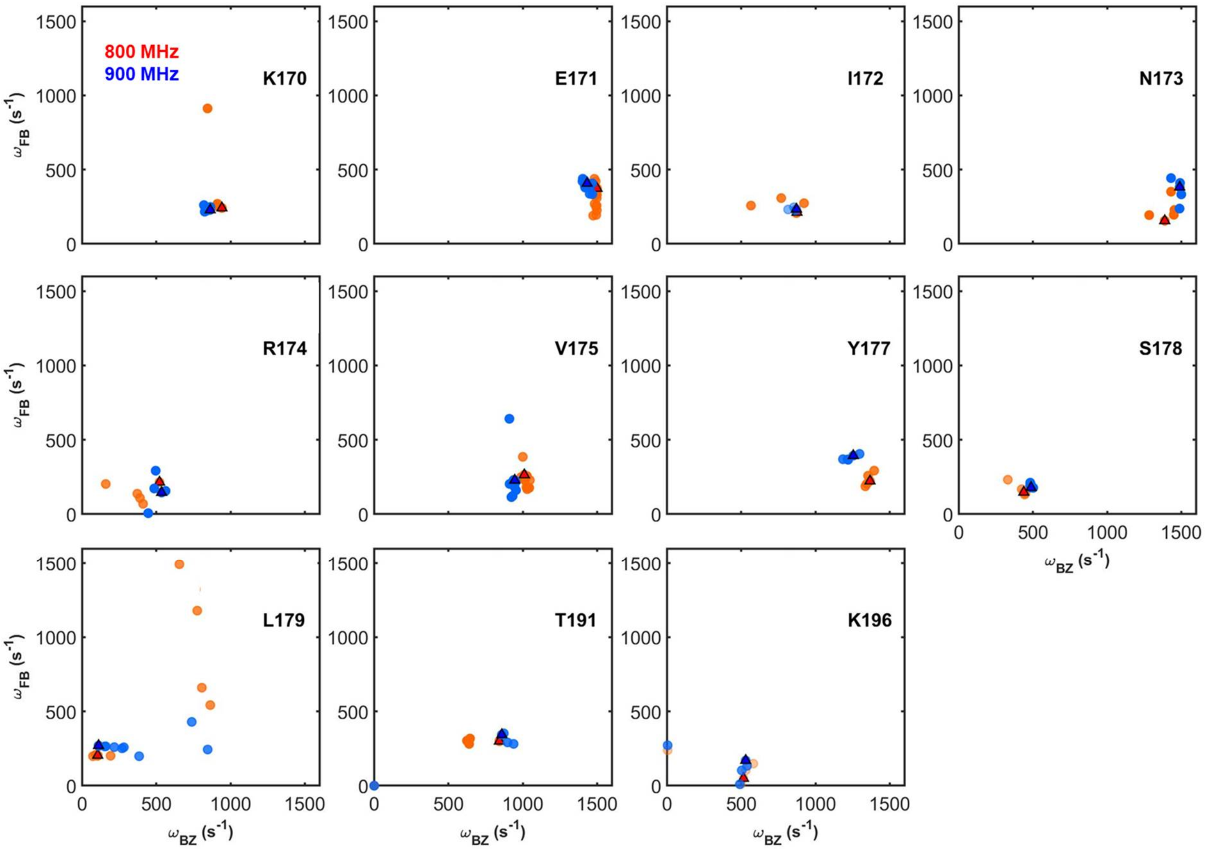

Figure S4. Residue dependent chemical shifts, ω_{FB} and ω_{BZ}, for each residue are numerically calculated based on single magnetic field basis GSIR. Global parameters are described by squares, while the optimized parameters of re-sampled results are shown with closed circles. All values of results are presented in Table S2. Results for 800 MHz and 900 MHz are indicated by red and blue colors, respectively.

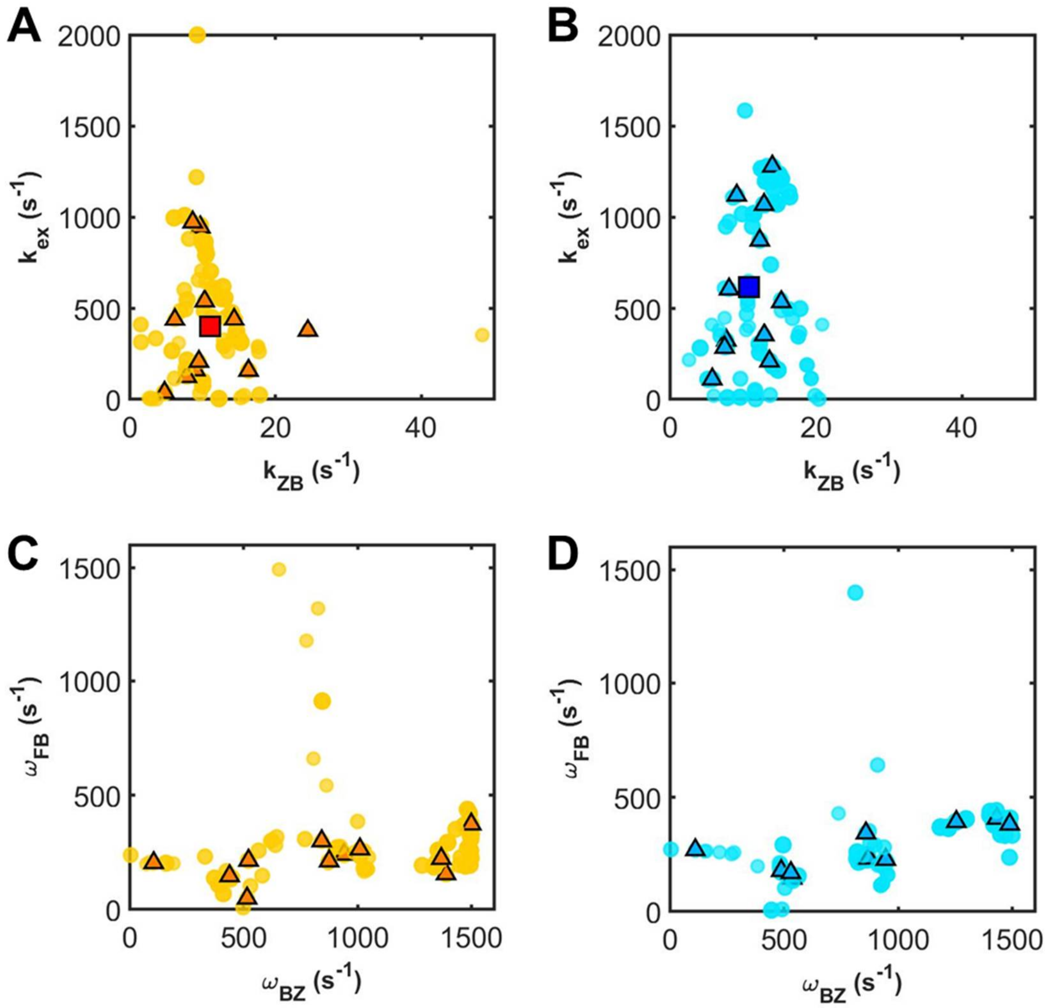

Figure S5. Residue dependent GSIR results are plotted for all residues: (A) rate constants at 800 MHz, (B) rate constants at 900 MHz, (C) chemical shifts at 800 MHz, and (D) chemical shifts at 900 MHz. Optimized rate constants and chemical shifts with re-sampled results (closed circles), global minima (triangles), and averaged values (for rate constants, squares) are plotted using different colors.

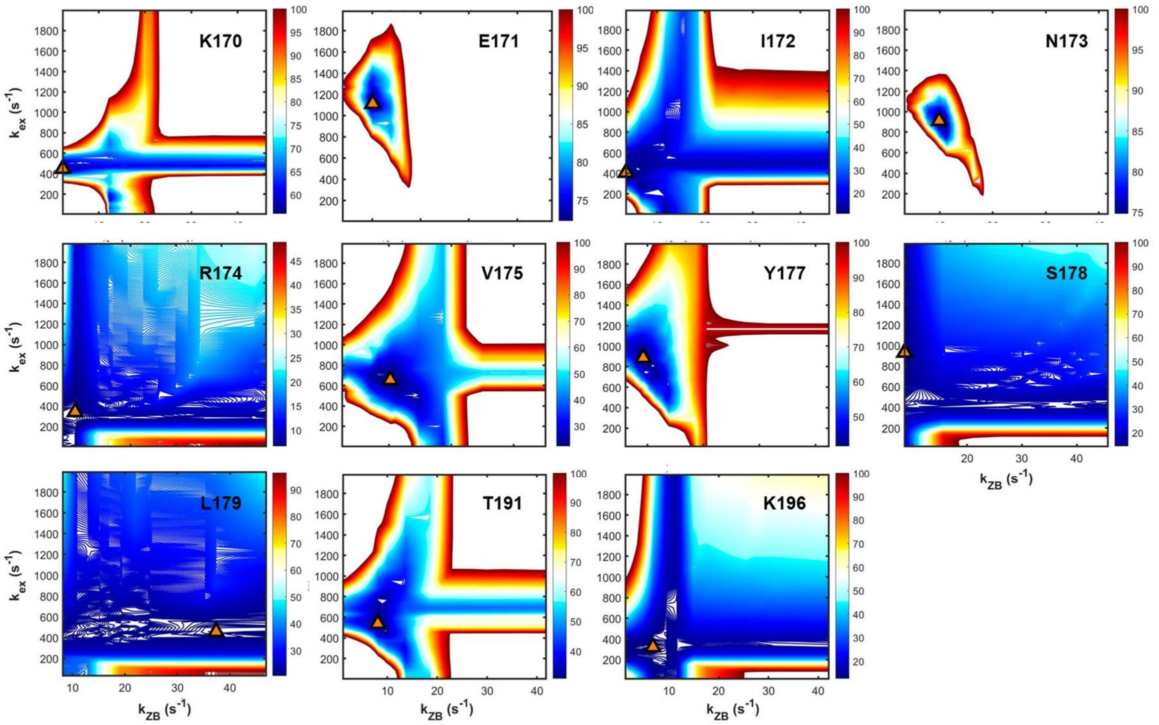

Figure S6. Global χ^2 map and minimum value for each residue are plotted. All χ^2 values are projected onto rate constants during GSIR analysis. Rate constants at global minimum are indicated by triangles.

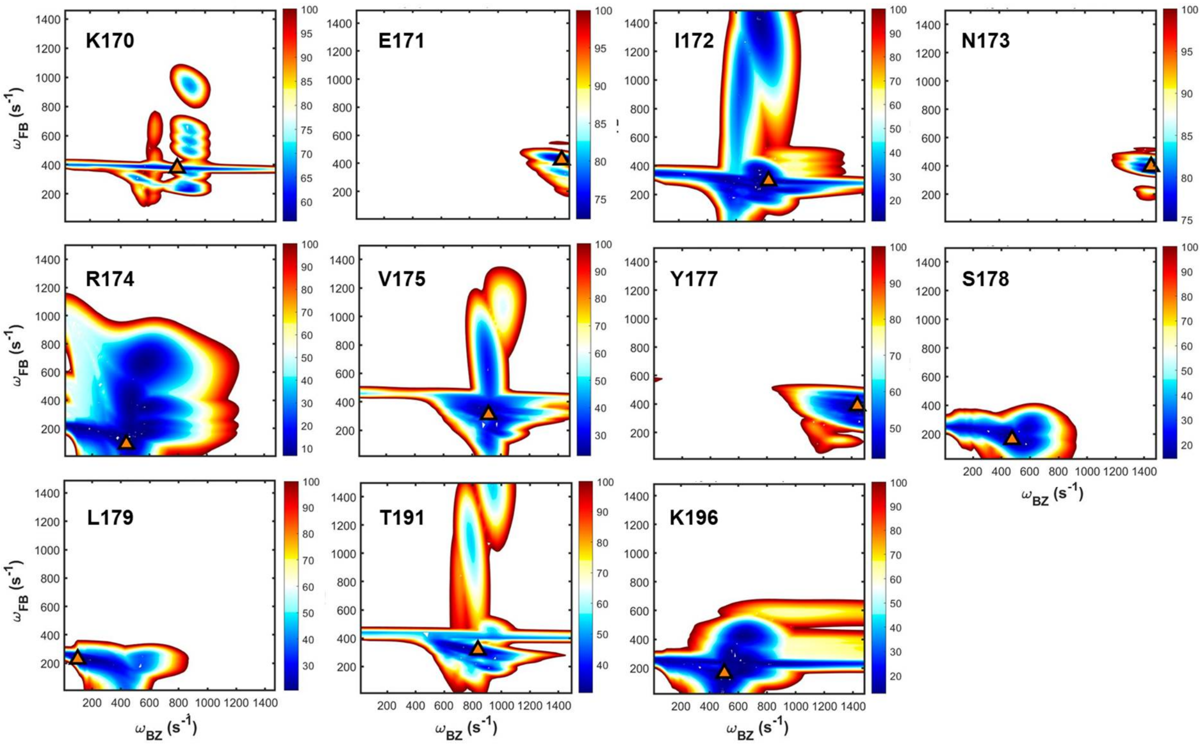

Figure S7. Global χ^2 map and minimum value for each residue are plotted. All χ^2 values are projected onto chemical shifts during GSIR analysis. Rate constants at global minimum are indicated by triangles.

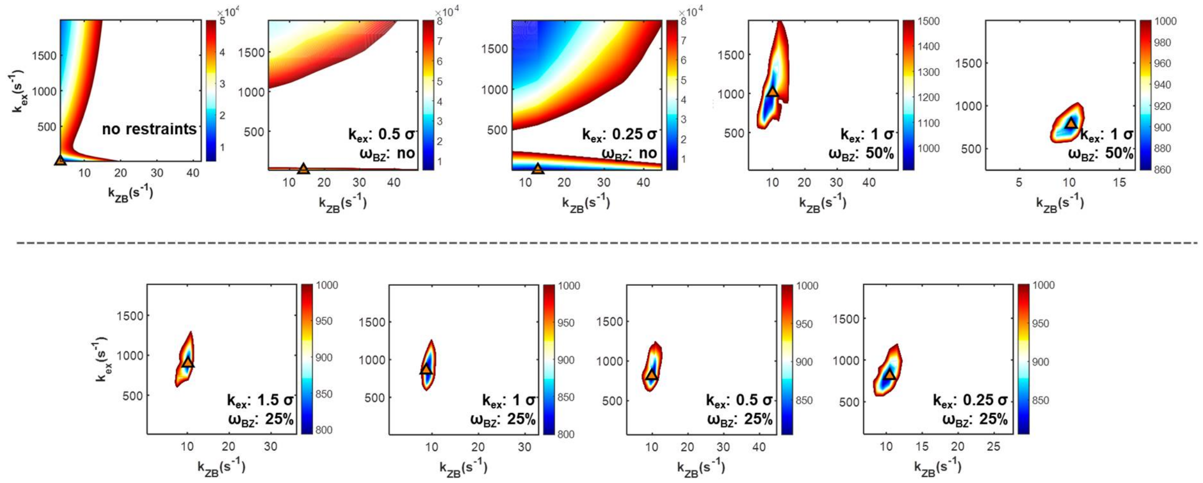

Figure S8. Global χ^2 maps and minima are plotted. All χ^2 values are projected onto rate constants during GSTR analysis with different restrained input parameters. Widths of Gaussian distribution are indicated. More details about restrained input parameters are described in the main text (Method Section). Rate constants at global minimum are indicated by triangles. The cases show large values and small values of minimum χ^2 are plotted at top and bottom, respectively.

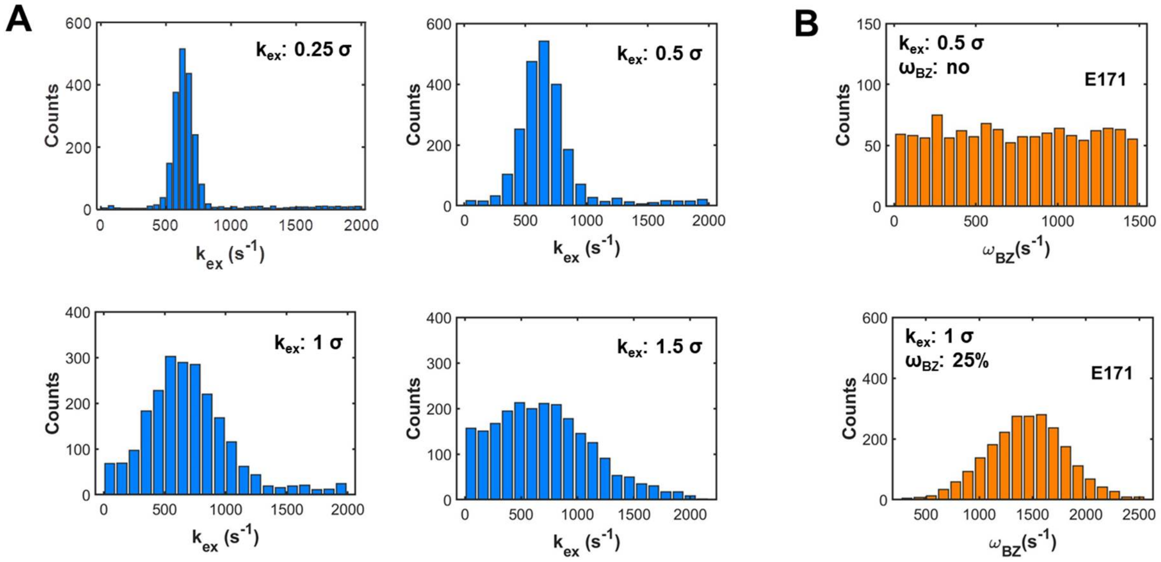

Figure S9. (A) Histogram of restrained input parameters for rate constant, k_{ex}, are plotted. (B) Representative histograms of restrained input parameters for chemical shifts of Glu171 are plotted. Widths of Gaussian distribution are indicated.

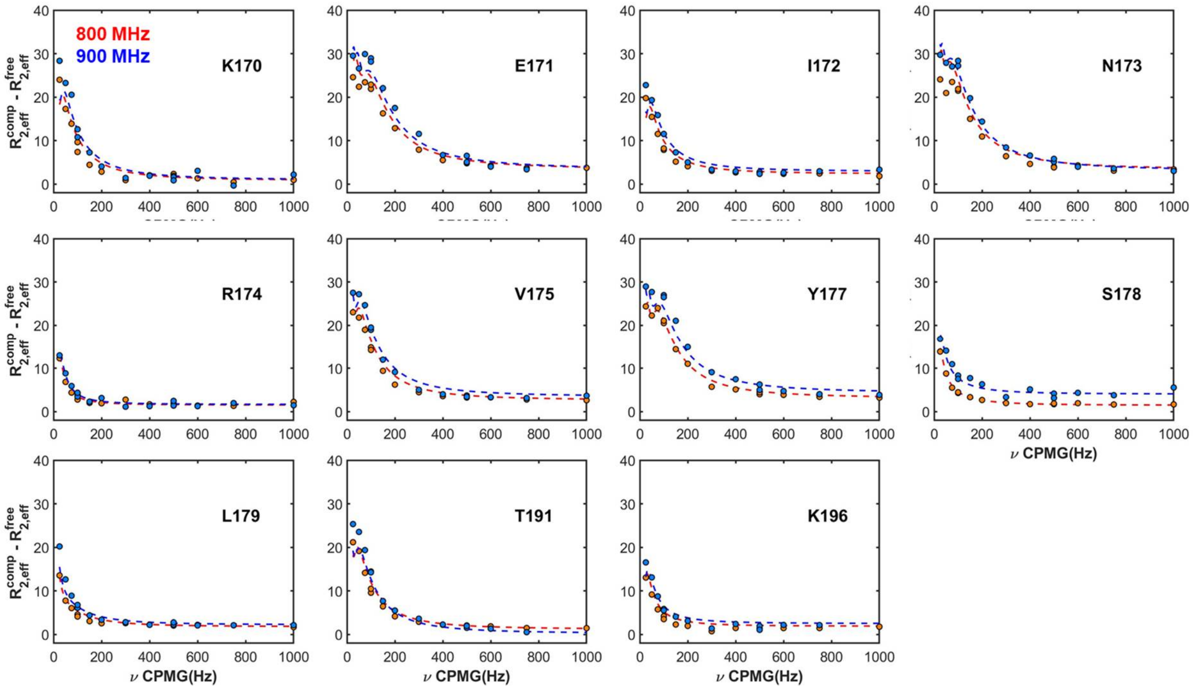

Figure S10. The ^{15}N CPMG relaxation dispersion data and R_2^{Calc} lines of global search for total residues (GFTR) of the d(CG)3-hZα_{ADAR1} complexes. The best GSTR results to Eq. 3 with 800 MHz and 900 MHz data are described by red and blue lines, respectively. Names of residues are denoted.

Table S1. Conformational exchange rate constants and chemical shift using fitting of two-state model at multi-magnetic field are presented.

	Individual Fitting		Global Fitting	
**parameter**	k_{ex} (s^{-1})	$\Delta\omega_{FC}$ (Hz)	k_{ex} (s^{-1})	$\Delta\omega_{FC}$ (Hz)
K154	252 ±50.6	112±5	832±55	143±59
A158	258 ±38	173±6		222±39
S162	256 ±18	189±3		242±36
K170	496 ±69	398±20		464±22
E171	1570 ±351	684±86		501±22
I172	512 ±44	349±11		404±23
N173	1310 ±264	619±63		505±21
R174	269 ±48	215±10		272±32
V175	792 ±98	472±24		480±21
Y177	1300 ±244	610±58		499±22
S178	378 ±70	250±15		307±29
L179	291 ±39	254±9		322±28
A180	276 ±30	180±5		231±38
Q186	255 ±22.6	174±4		223±39
A189	249 ±27.5	167±4		216±41
T191	635 ±66.3	417±17		458±22
W195	280 ±26.1	167±4		213±40
K196	361 ±44.4	260±10		322±28
I197	264 ±28.4	155±4		198±43

Table S2. Conformational exchange rate constants and chemical shift using single-field basis GSIR are presented. In order to compare, chemical shifts of 900 MHz cases were calibrated to 800 MHz field cases.

	800 MHZ				900 MHz			
**parameter**	k_{ex} (s^{-1})	$\Delta\omega_{FB}$ (Hz)	k_{ZB} (s^{-1})	$\Delta\omega_{BZ}$ (Hz)	k_{ex} (s^{-1})	$\Delta\omega_{FB}$ (Hz)	k_{ZB} (s^{-1})	$\Delta\omega_{BZ}$ (Hz)
K170	161	242	9.0	941	209	229	14	862
E171	946	373	10	1500	1280	408	14	1430
I172	125	212	7.8	875	324	234	7.7	871
N173	159	156	16	1390	874	382	12	1490
R174	37.9	215	4.8	521	111	146	5.8	535
V175	541	264	10	1010	537	228	15	946
Y177	440	223	14	1370	1070	393	13	1250
S178	974	147	8.6	438	1120	179	9.1	487
L179	378	205	24	105	353	270	13	111
T191	439	300	6.2	842	607	343	8.1	859
K196	208	49.4	9.5	515	285	170	7.5	530
